# Paradoxical Anxiety Level Reduction in Animal Chronic Stress: A Unique Role of Hippocampus Neurobiology

**DOI:** 10.3390/ijms23169151

**Published:** 2022-08-15

**Authors:** Vadim Tseilikman, Andrey Akulov, Oleg Shevelev, Anna Khotskina, Galina Kontsevaya, Mikhail Moshkin, Julia Fedotova, Anton Pashkov, Olga Tseilikman, Eduard Agletdinov, David Tseilikman, Marina Kondashevskaya, Evgenii Zavjalov

**Affiliations:** 1 School of Medical Biology, South Ural State University, 454080 Chelyabinsk, Russia; 2 Institute of Cytology and Genetics, Siberian Branch of the Russian Academy of Science, 630090 Novosibirsk, Russia; 3 Laboratory of Neuroendocrinology, Pavlov Institute of Physiology, RAS, 199034 St. Petersburg, Russia; 4 FSBI “Federal Neurosurgical Center”, Nemirovich-Danchenko Str. 132/1, 630087 Novosibirsk, Russia; 5 Department of Basic Medicine, Chelyabinsk State University, 454001 Chelyabinsk, Russia; 6 AO Vector-Best, Koltsovo Village, Research and Production Zone, Building 36, Room 211, 630559 Novosibirsk, Russia; 7 Zelman Institute of Medicine and Psychology, Novosibirsk State University, 630090 Novosibirsk, Russia; 8 Avtsyn Research Institute of Human Morphology, 117418 Moscow, Russia

**Keywords:** predator stress, anxiety, hippocampus, N-acetyl aspartate, phosphoryl ethanol amine, BDNF, lipid peroxidation

## Abstract

A paradoxical reduction in anxiety levels in chronic predator stress paradigm (PS) in Sprague–Dawley rats has recently been shown in previous works. In this paper, we studied the possible neurobiological mechanism of this phenomenon. We segregated PS-exposed Sprague–Dawley rats into the high- and low-anxiety phenotypes. The long-lasting effects of PS on corticosterone levels, blood flow speed in the carotid arteries, diffusion coefficient, and 1H nuclear magnetic resonance spectra in the hippocampus were compared in the high-anxiety and low-anxiety rats. In addition, we evaluated the gene BDNF expression in the hippocampus which is considered to be a main factor of neuroplasticity. We demonstrated that in low-anxiety rats, the corticosterone level was decreased and carotid blood flow speed was increased. Moreover, in the hippocampus of low-anxiety rats compared to the control group and high-anxiety rats, the following changes were observed: (a) a decrease in N-acetyl aspartate levels with a simultaneous increase in phosphoryl ethanol amine levels; (b) an increase in lipid peroxidation levels; (c) a decrease in apparent diffusion coefficient value; (d) an increase in BDNF gene expression. Based on these findings, we proposed that stress-induced anxiety reduction is associated with the elevation of BDNF gene expression directly. Low corticosterone levels and a rise in carotid blood flow speed might facilitate BDNF gene expression. Meanwhile, the decrease in apparent diffusion coefficient value and decrease in N-acetyl aspartate levels, as well as an increase in the lipid peroxidation levels, in the hippocampus possibly reflected destructive changes in the hippocampus. We suggested that in Sprague–Dawley rats, these morphological alterations might be considered as an impetus for further increase in neuroplasticity in the hippocampus.

## 1. Introduction

Chronic stress and high levels of glucocorticoids (GCs) produce functional and structural changes in the brain, particularly in the hippocampus, an important limbic structure that plays a key role in cognitive functions including learning and memory [1,2,3,4,5,6,7,8,9,10,11]. The hippocampus is a main plasticity brain region because it exhibits neuronal replacement, dendritic remodeling, and synapse turnover in response to numerous stress events [12,13]. According to McEwen, the “hippocampus became the gateway to understanding how systemic hormones affect higher brain functions” [14]. The hippocampus interacts with a variety of brain regions, including the prefrontal cortex, amygdala, and hypothalamus, to adjust anxiety levels in response to a variety of stressful conditions [15]. Chronic stress impairs hippocampus-dependent plasticity with a simultaneous increase in anxiety response [16].

Recently, it has been shown that on the fourteenth day after repeated exposures to predator scent stress, anxiety-like behavior was observed only in Wistar but not in Sprague–Dawley rats [17]. In turn, among the stressed Sprague–Dawley rats, some animals exhibited a lower level of anxiety-like behavior compared to other stressed animals and a control group. Notably, among the rats with an anxiolytic behavioral pattern, approximately 80% responded to the stressor in an active offensive manner [18]. On the other hand, the majority of rats with passive offensive reactions to stress were characterized by high anxiety levels [18]. Moreover, the active offensive rats had reduced plasma corticosterone [18]. The hippocampus is a glucocorticoid-responsive brain region [19]. Earlier, it has been demonstrated that a long-lasting increase in glucocorticoid concentration was a cause of dendritic shrinkage and loss of spines in the hippocampus that could be recognized as an event that disturbs plasticity [20]. High concentrations of glucocorticoids suppress the remodeling of hippocampal neurons and reduce their plasticity [14,21].

GCs exert numerous direct and indirect effects on the hippocampus [22]. They do so by acting on glucocorticoid (GRs) and mineralocorticoid receptors (MRs). GRs are widespread across the whole brain [23]. Contrarily, MR distribution is mainly restricted to the hippocampus [23]. MR and GR action as transcription factors is thought to underlie many responses to glucocorticoids [24,25]. Stress causes an increase in corticosterone which activates cytosolic glucocorticoid receptors [26,27,28]. The GR–GC complex translocates to the nucleus to modulate gene transcription, on the one hand, and to mitochondria to enhance mitochondrial oxidation, on the other hand [29]. It also accompanies the additional production of active oxygen species in the mitochondrial electron transport chain [30]. Subsequently, an increase in the production of superoxide, hydrogen peroxide, and hydroxyl radicals leads the cell to a state of oxidative stress which causes oxidative damage to DNA, protein carbonyl formation, and membrane lipid peroxidation (LPO) [30].

We hypothesized that the rise in LPO levels in the hippocampus might reflect the tissue integrity status. The apparent diffusion coefficient measured by magnetic resonance imaging might be considered a vital marker of neuronal integrity [31]. Overall, it causes apoptosis of hippocampal neurons. Notably, the brain-derived neurotrophic factor (BDNF) limited GC-induced oxidative stress in the hippocampus [32]. Some metabolites detected by ^1^H nuclear magnetic resonance spectra such as N-acetyl aspartate (NAA) and phosphoryl ethanolamine (PEA) are also considered markers of neuronal viability [32]. It is quite possible that indirect deleterious effects of GCs in the hippocampus might be associated with alterations in the brain blood flow rate. Recently, it has been reported that GC administration reduced the cerebral blood flow in the hippocampus and thalamus in dogs [33].

In light of these considerations, in this study, we examined whether low- and high-anxiety PS rats were associated with different corticosterone concentrations, rates of brain blood flow, alterations in NAA and PEA levels, free radical oxidation levels, and BDNF concentrations in the hippocampus.

## 2. Results

### 2.1. PS-Separated Sprague–Dawley Rats into Two Behavioral Phenotypes

For all rats subjected to chronic PS, the Kruskal–Wallis test did not reveal significant changes in time spent in the open arms of the elevated plus maze (EPM), in the time spent in the closed arms (X = 0.5, *p* = 0.74 for both parameters), in the number of entries in the open (X = 0.9, *p* = 0.52) and in the closed arms (X = 1.5, *p* = 0.38), and in the anxiety index (AI) value (X = 1.34, *p* = 0.42). However, significant differences were detected when PS rats were separated into behavioral phenotypes. Low-anxiety rats spent more time in the open arms of the EPM (*p* = 0.025) and less time in the closed arms (*p* = 0.025). The AI in low-anxiety rats was significantly smaller than that of control rats, whereas the AI of high-anxiety rats did not differ significantly from that of control rats (see Table 1).

### 2.2. Predator Scent Stress Reduced the Plasma Corticosterone Levels in Low-Anxiety Rats

**Figure 1:**
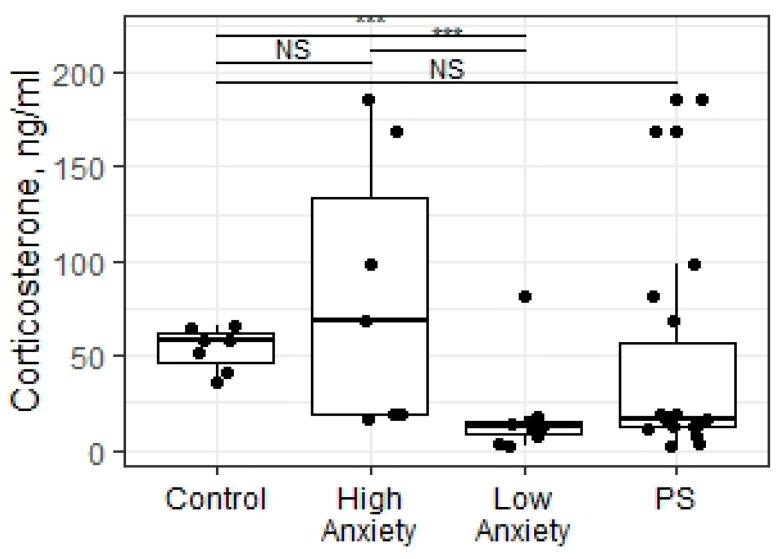
Plasma CORT concentrations (ng/mL) in PSS rats segregated into high- and low-anxiety groups according to AI as determined by performance in the EPM. Differences in plasma CORT concentrations among groups are shown as boxplots with dots representing individual data values and medians shown by horizontal lines. The boxes include the central 50% of the data, i.e., from the 25th to the 75th percentile. The whiskers include the data contained within 1.5 times the interquartile range. NS = *p* > 0.05; * *p* < 0.05; ** *p* < 0.01; *** *p* < 0.001. *p* values determined in non-parametric analysis.

The Kruskal–Wallis test revealed significant differences in the plasma corticosterone (CORT) concentrations in the rats exposed to PSS (X = 16.58; *p* = 0.0003; Figure 1). The CORT levels in low-anxiety rats were decreased compared to control and high-anxiety rats (*p* = 0.0027 and *p* = 0.0006, respectively). No significant differences in the plasma CORT levels were found between control and high-anxiety rats. We also found no differences in the plasma CORT concentration in the rats exposed to PSS compared to the control group (W = 91, *p* = 0.096 Mann–Whitney test). The Dunn test with Benjamini–Hochberg correction for multiple comparisons was used to assess pairwise statistical significance between groups after running the Kruskal–Wallis test.

### 2.3. Predator Scent Stress Increased the Blood Flow in Carotid Arteries in Low-Anxiety Rats

**Figure 2:**
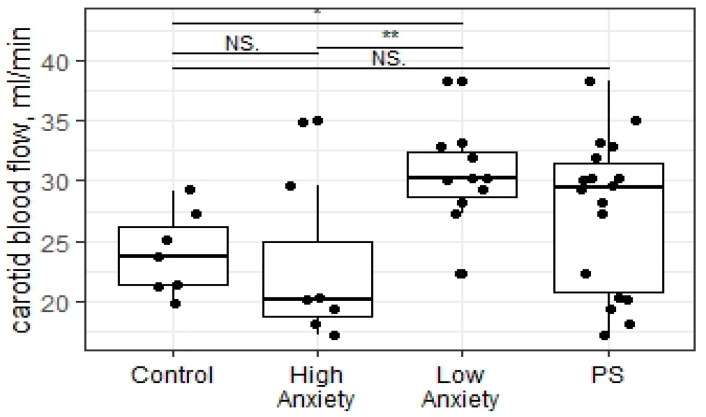
Carotid blood flow rate of PSS rats segregated into high- and low-anxiety groups according to AI as determined by performance in the EPM. Differences in blood flow rates among groups are shown as boxplots with dots representing individual data values and medians shown by horizontal lines. The boxes include the central 50% of the data, i.e., from the 25th to the 75th percentile. The whiskers include the data contained within 1.5 times the interquartile range. NS—non-significant, *p* > 0.05, * *p* < 0.05; ** *p* < 0.01. *p* values were determined in non-parametric analysis.

The Kruskal–Wallis test revealed significant differences in the volumetric blood flow of both carotid arteries in all experimental groups (X = 9.84; *p* = 0.01; see Figure 2). Low-anxiety rats showed significantly increased blood flow compared to high-anxiety and control animals (*p* = 0.008 and 0.016, respectively). There were no statistically significant differences in the blood flow in high-anxiety rats compared to the control group (*p* = 0.37). Moreover, the Mann–Whitney test did not return significant differences in the volumetric blood flow in both carotid arteries in the group subjected to PSS compared to the control (W = 39; *p* = 0.16).

### 2.4. Predator Scent Stress Reduced Apparent Diffusion Coefficient Values in the Hippocampus of Low-Anxiety Rats

**Figure 3:**
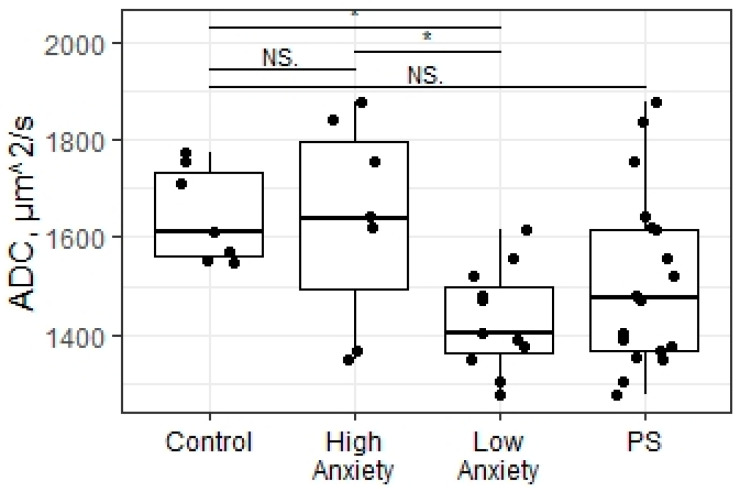
Apparent diffusion coefficient values of PSS rats segregated into high- and low-anxiety groups according to AI as determined by performance in the EPM. Differences in these values among groups are shown as boxplots with dots representing individual data values and medians shown by horizontal lines. The boxes include the central 50% of the data, i.e., from the 25th to the 75th percentile. The whiskers include the data contained within 1.5 times the interquartile range. NS—non-significant, *p* > 0.05; * *p* < 0.05. *p* values were determined in non-parametric analysis.

**Figure 4:**
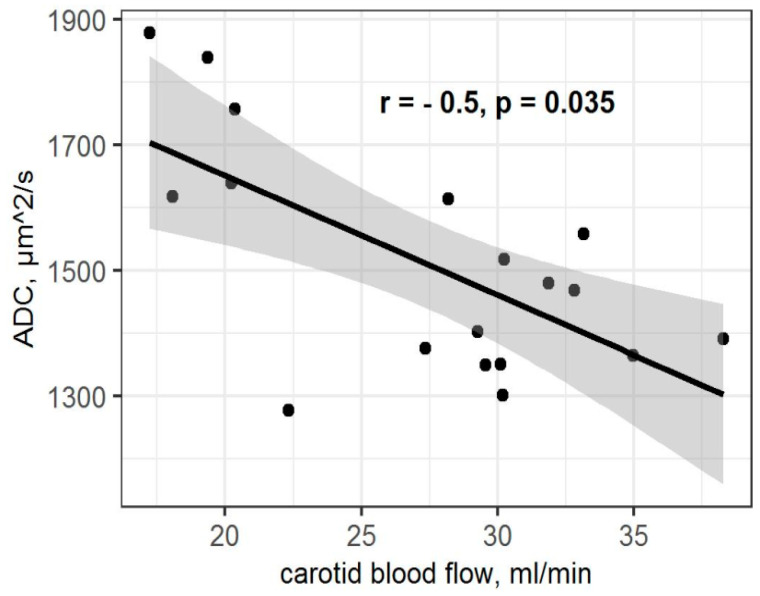
Spearman correlation between carotid blood flow rate and ADC values in PSS rats. Gray area around the line represents the 95% confidence interval.

**Figure 5:**
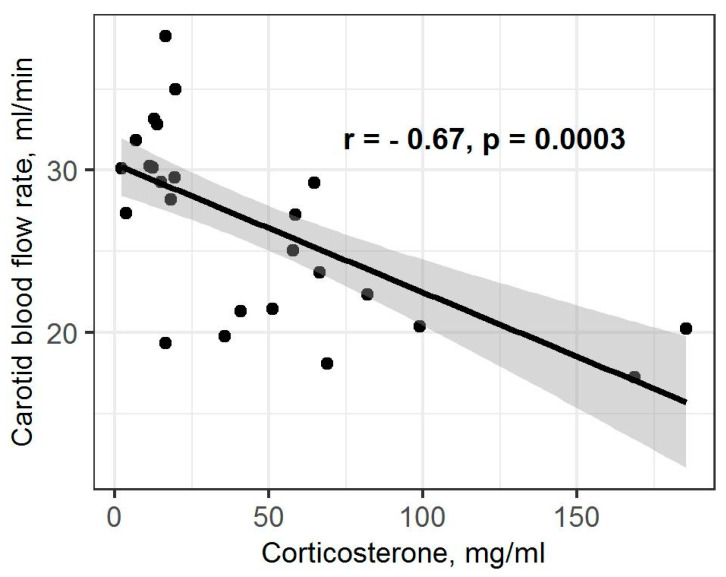
Spearman correlation between blood flow rate and plasma CORT levels in PSS rats. Gray area around the line represents the 95% confidence interval.

The statistical analysis found significant differences in the ADC values in the hippocampus between all the experimental groups of rats (X = 8.76; *p* = 0.01). The ADC values in the hippocampus of low-anxiety rats were significantly decreased compared to control animals (*p* = 0.016; Figure 3). The high-anxiety rats exhibited elevated ADC parameters compared to low-anxiety rats (*p* = 0.017). No significant differences between high-anxiety and control animals were found (*p* = 0.44). Despite the presence of significant differences among the high- and low-anxiety phenotypes, we found no differences in the ADC values in the hippocampus of the summarized sample of rats subjected to PSS compared to the control group of animals (W = 39; *p* = 0.16). Moreover, a significant correlation between ADC values in the hippocampus and the blood flow in both carotid arteries in PS-exposed rats (r = −0.5, *p* = 0.035; Figure 4) and between ADC values and corticosterone levels in stress-exposed rats (r = −0.67, *p* = 0.0003; Figure 5) were observed. However, we did not find a correlation between the ADC values in the hippocampus and the blood flow in both carotid arteries in high-anxiety rats.

### 2.5. Predator Scent Stress Altered 1H MR Spectra in the Hippocampus in High- and Low-Anxiety Rats

**Figure 6:**
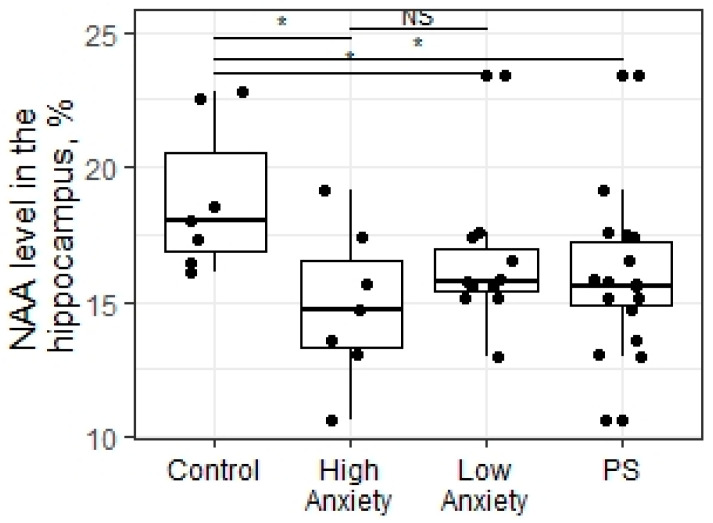
NAA level (%) in the hippocampus of PSS rats segregated into high- and low-anxiety groups according to AI as determined by performance in the EPM. Differences in NAA levels among groups are shown as boxplots with dots representing individual data values and medians shown by horizontal lines. The boxes include the central 50% of the data, i.e., from the 25th to the 75th percentile. The whiskers include the data contained within 1.5 times the interquartile range. NS—non-significant, *p* > 0.05; * *p* < 0.05. *p* values were determined in non-parametric analysis.

The Kruskal–Wallis test applied to the data revealed significant differences in N-acetyl-aspartate (NAA) levels in the hippocampus of all experimental groups (X = 6.53; *p* = 0.038, Figure 3). The decreased NAA levels in the hippocampus were detected in high-anxiety rats compared to control animals (*p* = 0.04). There also were significant differences in NAA levels in the hippocampus in low-anxiety rats compared to control animals (*p* = 0.05). We found no difference in the NAA levels in the hippocampus between high- and low-anxiety rats (*p* = 0.41). Correlation analysis did not find any significant relationship between the variables in all groups tested. The PSS did not change the NAA levels in the hippocampus compared to control animals (W = 91; *p* = 0.09; Figure 6).

**Figure 7:**
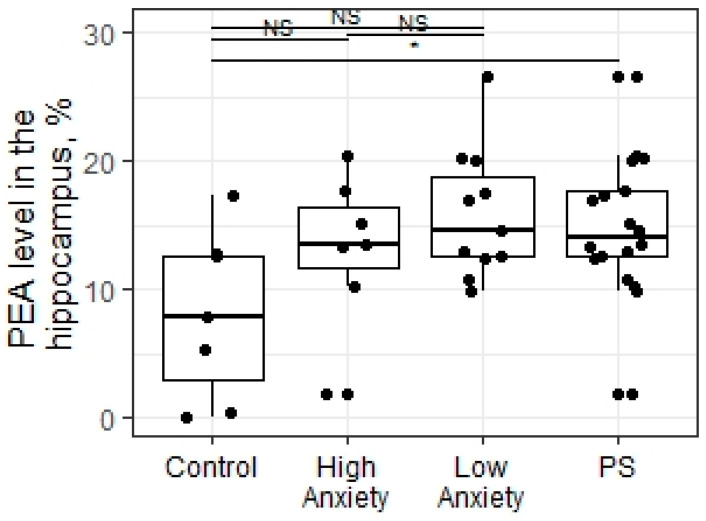
PEA levels (%) in the hippocampus of PS rats segregated into high- and low-anxiety groups according to AI as determined by performance in the EPM. Differences in PEA levels among groups are shown as boxplots with dots representing individual data values and medians shown by horizontal lines. The boxes include the central 50% of the data, i.e., from the 25th to the 75th percentile. The whiskers include the data contained within 1.5 times the interquartile range. NS—non-significant, *p* > 0.05; * *p* < 0.05. *p* values were determined in non-parametric analysis.

The statistical analysis (Kruskal–Wallis test) showed no significant differences in the phosphoryl ethanol amine (PEA) levels in the hippocampus between the experimental groups of rats (X = 5.39; *p* = 0.06; Figure 7). However, the PSS procedure changed the PEA levels in the hippocampus compared to control animals (W = 25; *p* = 0.02).

**Figure 8:**
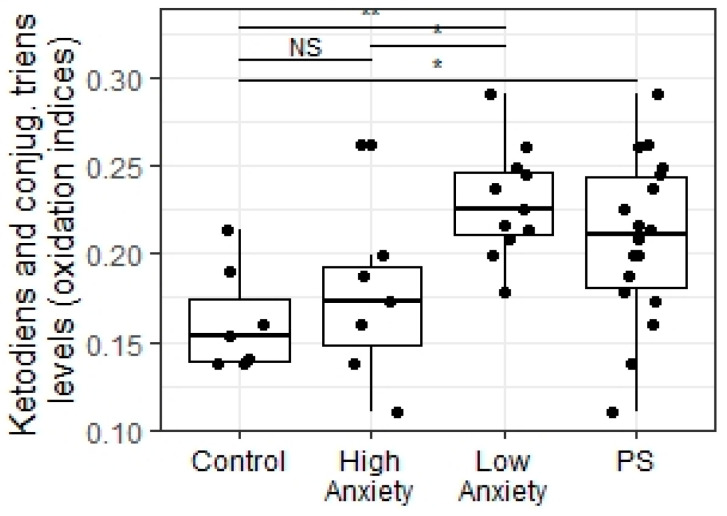
Ketodiens and conjugated triens levels (oxidation indices) in the hippocampus of PSS rats segregated into high- and low-anxiety groups according to AI as determined by performance in the EPM. Differences in ketodiens and conjugated triens concentrations among groups are shown as boxplots with dots representing individual data values and medians shown by horizontal lines. The boxes include the central 50% of the data, i.e., from the 25th to the 75th percentile. The whiskers include the data contained within 1.5 times the interquartile range. NS = *p* > 0.05; * *p* < 0.05; ** *p* < 0.01. *p* values were determined in non-parametric analysis.

The Kruskal–Wallis test detected a significant difference between experimental groups in the hippocampal level of propanol2-soluble ketodiens and conjugated triens (X = 11.42; *p* = 0.003; Figure 8). In the PS group, the concentration of ketodiens and conjugated triens was higher (W = 25.5, *p* = 0.025) than in non-stressed control rats. In low-anxiety rats, the hippocampal concentration of ketodiens and conjugated triens was also higher than in control rats (*p* = 0.006) and higher in comparison to high-anxiety rats (*p* = 0.019). In high-anxiety rats, the concentration of ketodiens and conjugated triens did not significantly differ from those of control rats (*p* = 0.61).

**Figure 9:**
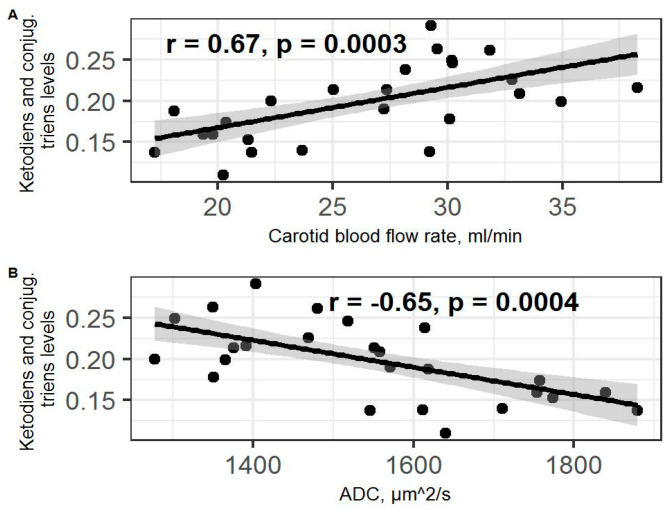
(**A**) Spearman correlation between blood flow values and ketodiens and conjugated triens level in PSS rats. (**B**) Spearman correlation between ADC values and ketodiens and conjugated triens level in PSS rats. Gray area around the line represents the 95% confidence interval.

A positive correlation between ketodiens and conjugated triens and blood flow rate in both carotid arteries (r = 0.67; *p* = 0.0003; Figure 9A) was observed. A negative correlation between ketodiens and conjugated triens levels and hippocampal ADC values (r = −0.65; *p* = 0.0004; Figure 9B) was found as well.

### 2.6. Predator Scent Stress Increased BDNF Gene Expression in Low-Anxiety Rats

**Figure 10:**
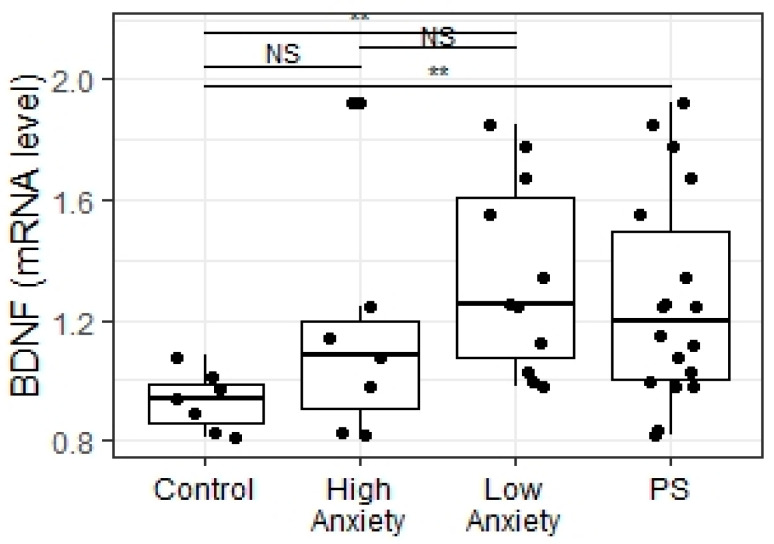
mRNA BDNF levels in the hippocampus of PSS rats segregated into high- and low-anxiety groups according to AI as determined by performance in the EPM. Differences in BDNF levels among groups are shown as boxplots with dots representing individual data values and medians shown by horizontal lines. The boxes include the central 50% of the data, i.e., from the 25th to the 75th percentile. The whiskers include the data contained within 1.5 times the interquartile range. NS—non-significant, *p* > 0.05; ** *p* < 0.01. *p* values were determined in non-parametric analysis.

Experimental groups significantly differed in hippocampal BDNF mRNA expression levels (X= 9.2; *p* = 0.01; Figure 10). Low-anxiety rats displayed higher BDNF mRNA levels than control animals (*p* = 0.008). There were no significant differences in the BDNF mRNA levels between high-anxiety rats and control animals (*p* = 0.22). We were able to detect a significant difference in BDNF mRNA expression levels between the PSS group and control animals (W = 19, *p* = 0.008).

**Figure 11:**
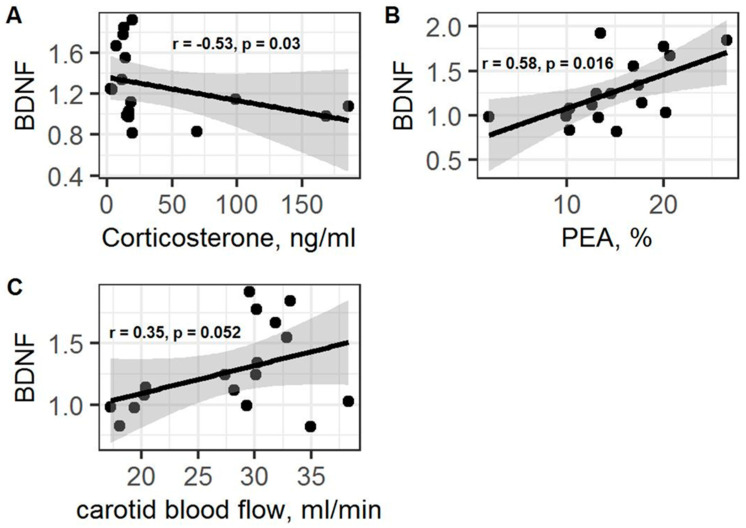
(**A**) Spearman correlation between BDNF and corticosterone levels in PSS rats. (**B**) Spearman correlation between BDNF and PEA values in PSS rats. (**C**) Spearman correlation between BDNF and carotid blood flow values in PSS rats. Gray area around the line represents the 95% confidence interval.

Positive correlations between BDNF gene expression and blood flow rate in both carotid arteries (r = 0.35; *p* = 0.052; with a tendency to statistical significance; Figure 11C), as well as a negative correlation between BDNF gene expression and plasma CS concentrations (r = −0.53; *p* = 0.03; Figure 11A), were observed. Moreover, a positive correlation between PEA levels and BDNF gene expression (r = 0.58; *p* = 0.016; Figure 11B) was also detected.

## 3. Discussion

The current study investigated Sprague–Dawley rats with different anxiety levels (low- and high-anxiety phenotypes). Ferguson and Cada have previously reported that anxiety-like behavior in the EPM is the most prominent in Sprague–Dawley rats strain compared to Wistar–Kyoto rats’ strain [34]. Our previous data on Wistar rats [35] together with the results of the present study confirmed that Sprague–Dawley rats are characterized by marked anxiety-like behavior.

The PS differently modified the AI of Sprague–Dawley and Wistar rats in the EPM. PS exposures increased the anxiety-like behavior in Wistar strain rats [35]. The AI of Sprague–Dawley rats strongly depended on the coping strategy in response to PS exposition [36]. The reduced anxiety-like behavior and lower plasma CS levels were registered in the rats with an active offensive response (AOR) to chronic PS compared to rats with a passive defensive offensive response (PDR). Interestingly, a recent study has demonstrated that stressed rats were divided into two behavioral phenotypes based on the immediate response of animals to a stressor [18,36]. In the present study, the long-lasting consequences of PS were taken as a specific indicator of behavioral phenotypes for the experimental groups of rats. We found that AOR- and low-anxiety rats had shown decreased anxiety-like behavior and low CS levels after PS exposures [18]. However, the percentage of AOR rats was significantly low, active rats were in the minority, and low-anxiety rats were the majority among the total number of stressed animals. Taking this into account, it might be assumed that a part of PDR rats had a low anxiety-like behavior after PS, whereas another part had not. Moreover, there was no similarity between AOR rats and low-anxiety rats in the patterns of ^1^HMR spectra in the hippocampus and striatum [36]. However, the main similarity between AOR rats and low-anxiety rats was in the fact that both of them had low CS levels. It is well in line with the findings of earlier studies [36]. A recent study indicated that chronic PS exposures were accompanied by a reduction in plasma CS levels in Wistar rats, and CS levels were decreased in low-anxiety rats more significantly than in high-anxiety rats [37]. This is also consistent with the results of other studies in Sprague–Dawley rats.

The absence of statistically significant results in the high-anxiety group may be explained by the fact that all measurements were performed 12–14 days post cessation of PS exposures. It is quite possible that significant changes in high-anxiety rats disappeared earlier. The conservativeness of high-anxiety rats might be connected to their metabolical state specifics. In our previous studies performed on Wistar rats [37], we also observed a lower variability in neuroendocrine changes in high-anxiety stressed rats compared with the low-anxiety group. At the same time, an interesting correlation was found between the level of anxiety and the level of microsomal oxidation, determined in vivo using the hexenal sleep test [37]. Additionally, animals with a long duration of hexenal sleep were considered slow metabolizers. It turned out that among highly anxious rats, the majority were slow metabolizers. Meanwhile, slow metabolizers are generally regarded as a metabolically conservative phenotype [37].

The elevation of carotid blood flow levels in low-anxiety rats is a principal and important finding of the current study. The negative correlation between AI values and carotid blood flow may be indicative of the presence of the link between behavior patterns and cerebral blood flow in PS rats. In this case, it is of special importance that the brain is extremely dependent on the delivery of oxygen, glucose, and other substrates from the blood [38]. Moreover, obtained data also point to the possible link between low GC levels and enhanced cerebral blood flow. This case is supported by a negative correlation between blood flow rate in both carotid arteries and plasma CS concentrations. It is in agreement with the reports demonstrating that GC administration can reduce CBF [39].

MRI analysis revealed an ADC value reduction in the hippocampus. Here, for the ADC array, we performed a diffusion weight images (DWI) procedure which is based on the calculation of H_2_O diffusion intensity across tissues [40]. A low intensity of H_2_O diffusion indicates a disruption in the tissue integrity [41]. Therefore, we propose that lower ADC levels could reflect disruption in the hippocampus integrity in low-anxiety rats.

It is commonplace that N-acetylaspartate (NAA) is considered a marker of neuronal viability [42]. NAA has repeatedly been implicated in many processes unfolding in the central nervous system (CNS). For example, it can be involved in the regulation of neuronal protein synthesis, myelin production, or metabolism of several neurotransmitters such as aspartate or N-acetyl-aspartyl-glutamate [42]. NAA reduction was synchronized with CS reduction in low-anxiety rats. Therefore, we take into account some reports, which demonstrate a strong positive correlation between cortisol and NAA levels in the hippocampus [43]. The hippocampus is one of the key players in the maintenance of stress resilience. Paradoxically, some damage-related markers in the hippocampus of low-anxiety phenotypes were observed, and it might be related to oxidative stress activation.

These suggestions are supported by our data reflecting the elevation of lypoperoxide levels in the hippocampus of low-anxiety rats. The CBF intensification might evoke oxidative stress in different brain areas, including the hippocampus. Notably, the concentration of lipoperoxides in the hippocampus is positively correlated with the blood flow rate in the carotid arteries. Moreover, the hippocampal lipoperoxide levels are negatively correlated with ADC values. In stark contrast to high-anxiety rats, it is not only the brain-structure-related processes but also an increase in the markers of neuron remodulation that were observed. BDNF is an essential neurotrophic factor for neuronal plasticity [44]. BDNF has different biological effects, such as preventing neuronal damage and death, improving neuronal pathological state, and promoting neuron regeneration. Recent studies have shown that elevated glucocorticoid levels impaired the BDNF expression in the hippocampus [45,46,47,48,49,50]. However, our data demonstrated the opposite results where the lower CS levels were associated with higher BDNF mRNA expression levels in the hippocampus of low-anxiety rats. It also should be noticed that in the hippocampus, MRs have a higher affinity for CS, compared to GRs (especially so in stress-resilient rats). Therefore, CS reduction might be considered in the context of optimization of hormone action limiting the deleterious effects of GCs on the hippocampus, including the suppression of BDNF production. The negative correlation between BDNF mRNA expression in the hippocampus and plasma CS concentrations supports these claims. We also observed a positive correlation between BDNF mRNA expression and PEA levels in the hippocampus. Meanwhile, among metabolites that were characterized using 1H-NMR spectroscopy, phosphorylethanolamine (PEA) was also a marker of the neuron’s viability. PEA is considered the most important precursor for the synthesis of sphingomyelin [51].

**Figure 12:**
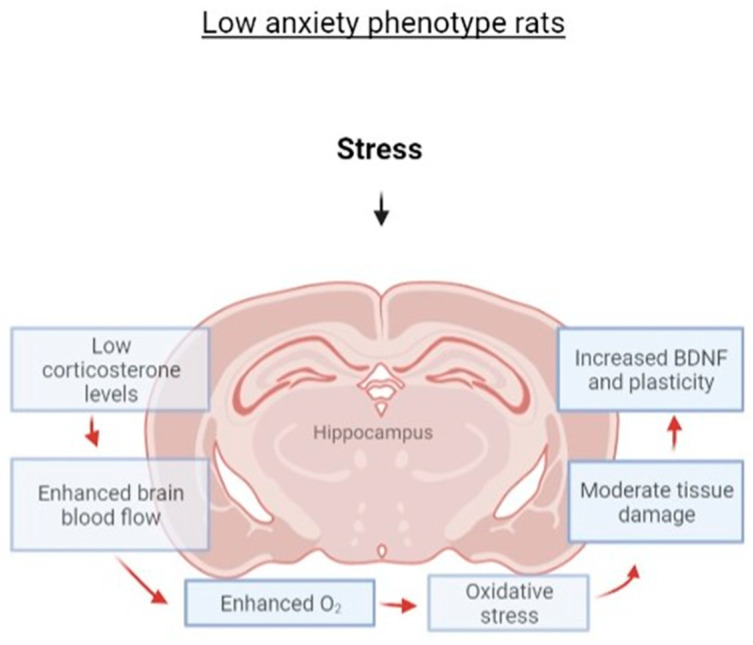
General scheme of hypothesized relations between stress exposure and hippocampal neurobiological characteristics.

In Figure 12, we summarized the main generalizations of the current study. Taken together, the elevation of hippocampal BDNF gene mRNA expression as well as the increase in PEA levels in low-anxiety rats might be considered markers of resilience to PS exposures. Presumably, primary destructive alterations in the hippocampus led to secondary protective effects in a phenotype-specific manner. Nowadays, it is well known that the activation of free radical oxidation is a side effect of increased oxygen consumption [52]. Some data suggested that hyperoxygenation treatment increased the expression of BDNF through the upregulation of MeCP2/p-CREB activity in the hippocampus of mice [53]. Thus, the study revealed new associations between anxiety behavior and neurobiological changes in the hippocampus in PS-exposed Sprague–Dawley rats.

## 4. Methods

### 4.1. Experimental Procedure

For the PSS protocol, rats were exposed to cat urine scent in a Petri dish with litter for 10 min daily for 10 days (21 rats were submitted to stress exposure; 8 control rats were exposed to a neutral scent). Repeated exposure to the PSS may be a more accurate model of human PTSD than a single acute exposure approach, granted that it minimizes the effect of confounding factors, such as the concentration of pheromones in each individual urine scent exposure [54]. All procedures were performed between 1:00 and 2:00 p.m. During the scent exposure protocol, stress-related behavior was captured daily via a web camera. Behavioral evaluation was conducted via the 3D animal tracking system “EthoStudio” [18,54]. The evaluator of the behavior had not previously worked with any rats in our groups. Recorded variables included the time spent in the open and closed arms of the maze and the number of entries into the open and closed arms.

**Figure 13:**
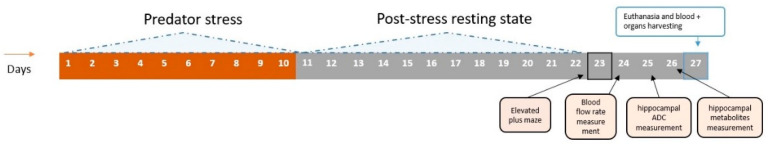
Graphic timeline of the experiment.

The timeline for modeling PSS, evaluating stress-related behavior and anxiety, and measuring metabolites (CORT, Glu + Gln, and 11-dehydrocorticosterone) in plasma, brain, and adrenal glands, respectively, was as follows (Figure 13):

1. Days 1–10: PSS;
2. Days 11–22: rest;
3. Day 23: elevated plus maze test;
4. Day 24 blood flow rate in carotid artery measurement by MRA;
5. Day 25 hippocampal apparent diffusion coefficient measurement by MRI;
6. Day 27: hippocampal metabolite measurement by MRS;
7. Day 28: euthanasia, harvesting of blood and organs.

### 4.2. Behavioral Activity

Video recordings of PSS sessions were made in the home cages. The presence or absence of behavioral responses was recorded daily. The frequencies of freezing, grooming, sniffing of stimuli, climbing on stimuli, and tearing of protective cover of stimuli were used for the classification of rats as AFR and APR. The presence of the response in one session was marked with “1”, while the lack of a response was marked as “0”. Apart from the registration of the daily changes in the observed behavioral responses, we also summarized the frequencies of these behavioral responses over 10 days. The predator stress outcome was evaluated with an elevated plus maze test, using the standard elevated plus maze (EPM) test apparatus TS0502-R3 (OpenScience, Moscow, Russia). Variables recorded included time spent in the open and closed arms of the maze and the number of entries into the open and closed arms.

The identification of the high- and low-anxiety phenotypes among the rats exposed to predator scent stress: 

The anxiety level of the rats exposed to predator stress was tested by the elevated plus maze (EPM) as published previously [54,55]. On the next day after 14 days of the post-stress period, the rats were submitted to the EPM test. The following parameters were registered: (1) the number of entries into the open arms; (2) the number of entries into the closed arms; (3) the time spent in the open arms; (4) the time spent in the closed arms.

Based on these measurements, an anxiety index (AI) was calculated: AI = 1 − {[(time into the open arms/time on maze) + (number of entries into the open arms/number of all entries)]/2}. The behavior was recorded for 300 s in the EPM apparatus using a video system SMART with SMART 3.0 software. The equipment was cleaned in between sessions. AI discriminant of 0.8 was set according to our previous study [55]. The accuracy of prediction of a rat’s belonging to the high- or low-anxiety subgroup, calculated by the canonical discriminant analysis of the behavioral data sets obtained from control and PS rats with an AI discriminator set at 0.8, was 100% in control and 38% in PS rats.

### 4.3. Magnetic Resonance Imaging

MRI was performed via a horizontal tomograph with a magnetic field of 11.7 tesla (Bruker, Biospec 117/16 USR, Ettlingen, Germany). All rats were anesthetized with gas (isoflurane; Baxter Healthcare Corp., Deerfield, IL, USA) using a Univentor 400 Anesthesia Unit (Univentor, Zejtun, Malta). The tomographic table contained a water circuit that maintained a surface temperature of 30 °C to preserve animal body temperature during the test. A pneumatic respiration sensor (SA Instruments, Stony Brook, NY, USA), placed under the lower body, controlled the depth of anesthesia. MRI was recorded with transmitter volume (T11232V3) and rat brain receiver surface (T11425V3) using 1 Hz radiofrequency coils (Bruker, Ettlingen, Germany). High-resolution T2-weighted images of the rat brain in three (axial, sagittal, and coronal) dimensions (section thickness, 0.5 mm; field of view, 2.5 × 2.5 cm for axial and 3.0 × 3.0 cm for sagittal and coronal sections; matrix of 256 × 256 dots) were recorded by rapid acquisition with relaxation enhancement (TurboRARE), with the pulse sequence parameters TE = 11 ms and TR = 2.5 s.

To reduce motion artifacts, there were several techniques that we used: (1) Using a sufficient concentration of isoflurane in the air mixture during gas anesthesia of 1% and above. The content of isoflurane in the air mixture during the scanning session was maintained at a level of 1.5% at a flow of 300 mL/min. (2) Control of breathing (depth and purity of respiratory movements) of the animal and monitoring of its movement. Using a pneumatic sensor, which is included in the MRI system, we carried out such observations on the animal during scanning. (3) Sufficiently reliable fixation. We used the fastening systems that the manufacturer of the MRI bed produced, namely: fastening the anterior incisors to the bed and rigid fixators in the area of the ear bones. (4) A software algorithm that reduces motion artifacts. We used the “motion suppression” software option for DWI which is incorporated by the MRI scanner manufacturer into the ParaVision 5.1 software package.

### 4.4. Magnetic Resonance Angiography

**Figure 14:**
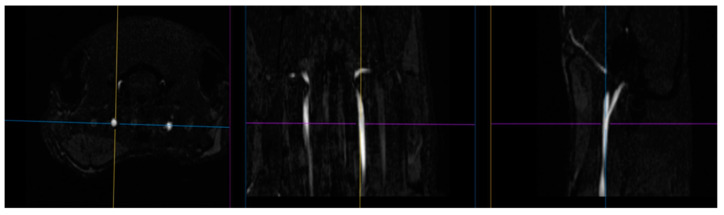
The images of the carotid arteries in three projections (in order to select the area of blood flow assessment).

**Figure 15:**
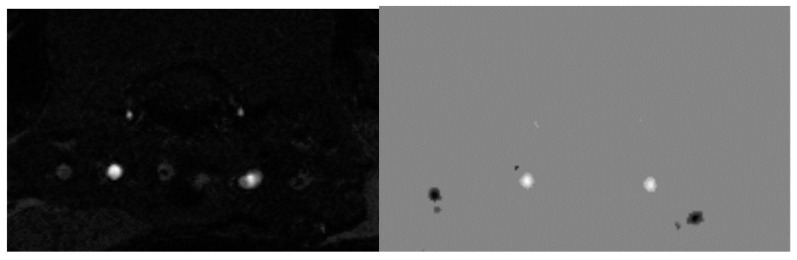
(**Left**) 2D TOF image of carotid arteries; (**right**) 2D image of the same area obtained using PCA.

The blood supply of the brain was examined using time of flight (TOF) angiography in one scan session with MRI [56,57]. Images of the common carotid arteries (left and right; Figure 14) were obtained in a two-dimensional projection (an example is shown in Figure 15), which, by means of the ParaVision5.1 MIP (maximum intensity projection) option, were converted into 3D images. The measurement of the size of the vessels was performed using an ROI placed 2 mm proximal to the bifurcations of the carotid arteries, which were determined from 3D images. Along with the imaging of angioarchitectonics, the blood flow velocity was assessed by the method of phase contrast angiography PCA (phase contrast angiography). The measurement was performed in a single cross-sectional main blood flow section, also guided by a 3D model (an example of PCA phase contrast angiography is shown in Figure 2). We performed these measurements 2 mm proximal to the bifurcations of the carotid arteries, that is, in full accordance with the area of measurement of dimensions of the vessels’ lumen. Additionally, the maximum blood flow velocity in the central part of the artery and the average linear blood flow velocity for the entire section of the lumen of the artery were determined. Volumetric blood flow (mL/min) was calculated based on the mean blood flow velocity and cross-section. All hemodynamic characteristics were obtained in a 2 min recording interval with averaged values as a result of several complete heartbeat cycles. The following indicators were used in the paper: the speed of blood flow in the left and right common carotid arteries (velocity of OSA L, cm/s; velocity of OSA P, cm/s); the area of the lumen of the left and right common carotid arteries (lumen o.s.a. L, mm^2^; lumen o.s.a. P, mm^2^); volumetric blood flow of the left/right common carotid artery and their sum score (o.s.a. blood flow L, mL/min; o.s.a. P blood flow, mL/min; o.s.a. blood flow, mL/min).

### 4.5. Diffusion-Weighted Magnetic Resonance Imaging

**Figure 16:**
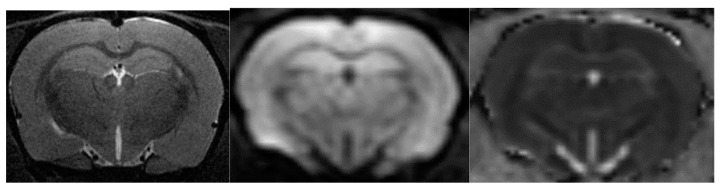
From left to right are images of T2-WI, DWI, ADC.

As with MRA, DWI was performed in one scan session with two other MRI techniques [58]. To obtain diffusion-weighted tomograms, a three-dimensional echo-planar pulse sequence was used with the following parameters: TE = 15.4 ms; TR = 2000 ms; the amount of excitation is 1, and the isotropic voxel size is 200 µm. The data were obtained using a multi-pass acquisition with five directions and three repetitions for b = 0, 100, 200, 500, 1000 s/mm^2^. DWI data processing with measured diffusion coefficient (ADC) estimation was performed using ParaVision 5.1, Image Sequence Analysis Tool, dtraceb function from Bruker sequence analysis library to generate average ADC for each ROI based on the equation: ADC = ln (S0/Sn)/bn, where S0 is an intensity of the T2-weighted image (b = 0), and Sn is an intensity of the diffusion-weighted image with bn as the decay factor of the diffusion gradient. This data processing step resulted in a parametric map of the numerical values of the measured diffusion coefficient. Eight brain slices with calculated ADCs were obtained; they anatomically corresponded to T2-weighted images in the Bregma range: from 1.2 mm to −3.4 mm). Please see Figure 16 for corresponding brain images.

To correct for apparent translational motion caused by frequency drift and gradient diffusion, all slices were aligned using T2-weighted images and by strict registration orientation. The measured diffusion coefficient was assessed in the hippocampus (HPF).

Due to the initially low signal and complex processing during the implementation of the DWI protocol, multiple error reduction techniques are needed. In particular, hardware- and physiology-induced artifacts are of great importance: movements, heartbeat, respiration, temperature effects. The DWI protocol that we used is not so sensitive to artifacts, partly due to the higher level of the original MRI signal, and also does not require complex image post-processing algorithms (we used the ParaVision5.1 software package with the ADC calculation function supplied by the Bruker MRI system manufacturer).

### 4.6. Magnetic Resonance Spectroscopy (MRS)

We performed the analysis of rat dorsal hippocampus neurometabolites following the execution of the abovementioned methods [57]. 

Voxels were manually placed according to a structural T2-weighted MRI image. All proton spectra were recorded by spatially localized single-voxel stimulated echo acquisition mode (STEAM) spectroscopy, with the following pulse sequence parameters: TE = 3 ms, TR = 5 s, and 120 accumulations. Uniformity of the magnetic field was tuned within the selected voxel using FastMap before each spectroscopic recording. The water signal was inhibited with a variable pulse power and optimized relaxation delays (VAPOR) sequence. The experimental 1H magnetic resonance spectra were processed, and the quantitative composition of metabolites was determined with a custom-made program similar to that of the LC Model software package [59,60]. The baseline correction was conducted automatically by the program to determine the spectral baseline for fitting the spectrum obtained by 1H MRS. The process of fitting was presented on the real-time plot, and the fitted data were stored in numerical form.

**Figure 17:**
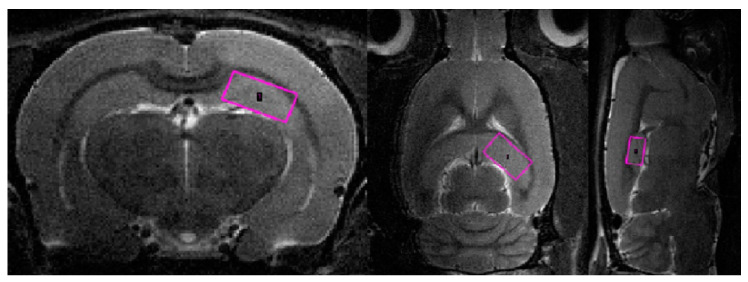
The regions of the MRS spectrum registration in three orthogonal planes of the brain of studied rats.

The facilities of the program allow the following 12 brain metabolites to be fitted to the MRS spectrum: N-acetylaspartate (NAA); phosphorylethanolamine (PEA); choline compounds (Cho); creatine + phosphocreatine (Cr + PCr); myo-inositol (mIno, Ins); alanine (Ala); lactate (Lac); glutamate + glutamine (Glu + Gln); aspartate (Ast); γ-aminobutyric acid (GABA); glycine (Gly); and taurine (Tau). The percentage ratios of metabolites were analyzed (Supplemental Table S1). Please see Figure 17 for corresponding images.

### 4.7. Hormonal Measurements

Between 11:00 a.m. and 1:00 p.m. on experimental day 28, rats were sacrificed by decapitation, and blood samples were collected in tubes with heparin. Blood samples were then centrifuged at 3000 rpm at 4 °C for 15 min. Plasma samples were aliquoted and stored in a −80 °C freezer until the required use. After thawing, plasma CORT concentrations were measured with ELISA (Cusabio ELISA Kit, Houston, TX, USA) as per manufacturer’s instructions. The assay sensitivity was 0.25 ng/mL, and the intra- and inter-assay coefficients of variation were both <5%.

### 4.8. Evaluation of Oxidative Stress

Hippocampal level of lipid peroxidation products. The tissue level of lipid peroxidation products was assayed by an extraction, spectrophotometric method. This method allows differential measurement of acyl peroxides among phospholipids extracted from propanol-2 phases along with non-esterified intermediates of fatty acid peroxides extracted from the heptane phase. Results were expressed as oxidation indices: E232/220 for relative levels of conjugated dienes, E278/220 for ketodiens, and conjugated triens [61].

### 4.9. BDNF Gene Expression in the Hippocampus

Total cellular RNA was isolated using a guanidine isothiocyanate method. A total of 1 µg of total RNA was reverse transcribed with the 100U MMLV Reverse Transcriptase (Sibenzyme, Novosibirsk, Russia), 1 mM dNTP, 2 mM DTT, 2 µM OligodT primer (Evrogen, Moscow, Russia), and standard thermocycler temperature conditions for MMLV. All real-time PCR reactions were performed using the ABI ViiA7 system (Thermo, Waltham, MA, USA) and standard cycle. Amplifications were performed using the real-time PCR Master Mix qPCRmix-HS  +  LowROX (Evrogen) and primers and Taqman probes. Using validated TaqMan primer probes for BDNF (Thermo ScientificRn02531967_s1), cDNA was run in triplicate and analyzed using the 2^−ΔΔCT^ method and normalized to the β-actin housekeeping gene (Thermo Scientific Rn00667869_m) as an arbitrary unit.

### 4.10. Data Analyses

Data were analyzed with SPSS 24 (IBM, New York, NY, USA), STATISTICA 10.0 (StatSoft, Tulsa, OK, USA), Rstudio (RStudio, Boston, MA, USA), and Excel (Microsoft, Redmond, WA, USA) software. The normality of data distributions was examined with the Shapiro–Wilk test. Non-normally distributed data were analyzed with a nonparametric, one-factor Kruskal–Wallis ANOVA followed by Dunn tests for pairwise comparisons between respective groups. Relationships between variables were examined by Spearman correlation analysis. *p* < 0.05 was considered significant.

## 5. Conclusions

Chronic PS led to multiple long-lasting behavioral and neurobiological alterations in rats. All observed alterations such as a reduction in plasma corticosterone levels, elevation of blood flow levels, decrease in ADC values and NAA levels with a simultaneous increase in the PEA levels, and an elevation of the BDNF gene expression in the hippocampus were observed only in the low-anxiety phenotype. In rats belonging to the high-anxiety phenotype, there were no alterations found in any neurobiological and endocrine parameters. Moreover, PS exposure did not lead to anxiety behavior alterations for this phenotype. Meanwhile, Sprague–Dawley rats had more significant anxiety levels than Wistar rats, which sustained PS exposures earlier. Thus, based on the detected behavioral, neurobiological, and endocrine parameters, we suppose that high-anxiety rats are conservative, whereas low-anxiety rats are reactive respondents in relation to PSS exposures. In turn, the responsiveness of the low-anxiety phenotype includes the sensitivity of the cerebral blood flow and hippocampus, on the one hand, and the sensitivity of the plasma corticosterone levels to the PSS exposure, on the other hand. The hippocampus is a glucocorticoid-sensitive brain region. The negative correlations between plasma corticosterone and some hippocampal neurobiological parameters are well in accordance with this fact.

## 6. Limitations

It is a pilot study where the integrative estimation of cerebral blood flow was the first priority. Unfortunately, we ignored the opportunity to directly array blood flow in the hippocampus, although the arterial spin labeling MRI method allows this. Therefore, in the next study, we will directly measure the blood flow in the hippocampus.

## Figures and Tables

**Table 1:** Results of EPM behavioral experiments.

	Control (*n* = 7)	PS (*n* = 18)	High Anxiety (*n* = 11)	Low Anxiety (*n* = 7)
Time spent in open arms (s)	45 ± 14	82 ± 9	60 ± 12	103 ± 7 * #
Time spent in closed arms (s)	565 ± 14	518 ± 15	450 ± 14	497 ± 7 * #
Entries to open arms	2.1 ± 0.8	2.5 ± 0.6	1.7 ± 0.2	4.4 ± 0.4
Entries to closed arms	12.9 ± 1.7	9.5 ± 2.01	7.6 ± 0.6	9.0 ± 0.7
AI (anxiety index)	0.89 ± 0.02	0.82 ± 0.27	0.86 ± 0.01	0.75 ± 0.03 ** #

Data are the mean ± SEM. Different from control * *p* < 0.05, ** *p* < 0.01. Different from high-anxiety phenotype, # *p* < 0.05. AIs of high- and low-anxiety rats differed a priori and were not compared statistically.

## Data Availability

Data available upon reasonable request.

## Supplementary Materials

The supporting information can be downloaded at: https://www.mdpi.com/article/10.3390/ijms23169151/s1.
