# Supplementary material for: Paradoxical Anxiety Level Reduction in Animal Chronic Stress: A Unique Role of Hippocampus Neurobiology
Source: Int J Mol Sci. 2022 Aug 15;23(16):9151. doi: 10.3390/ijms23169151 (PMC9409467; doi:10.3390/ijms23169151)
Supplement: Supplementary file 1 [file ijms-23-09151-s001.zip › ijms-1839820-supplementary.pdf]

Supplementary Materials

Table S1. Individual metabolite levels (expressed as a percentage of the total amount of all metabolites) in hippocampus of PS exposed rats.

**Metabolite ratios in hippocampus (%)**	**Control (*n* = 7)**	**PS (*n* = 18)**	**High Anxiety (*n* = 7)**	**Low Anxiety (*n* = 11)**
**NAA**	18.8 ± 2.75	15.85 ± 2.73*	14.89 ± 2.84*	16.46 ± 2.6*
**GABA**	7.35 ± 3.25	6.71 ± 2.14	5.6 ± 1.36	7.61 ± 2.28
**Ala**	7.03 ± 3.65	5.82 ± 2.6	7.41 ± 0.77	5.35 ± 1.85
**Ast**	0.1 ± 0.02	0.09 ± 0.01	0.11 ± 0.04	0.081 ± 0.05
**Cho**	2.17 ± 0.85	1.88 ± 0.44	1.96 ± 0.45	1.78 ± 0.31
**Cr + PCr**	12.00 ± 1.19	10.00 ± 2.35	10.99 ± 0.55	9.78 ± 0.58
**Glu + Gln**	15.01 ± 4.66	13.59 ± 4.61	12.90 ± 1.47	14.43 ± 1.83
**mIn**	5.46 ± 1.64	3.95 ± 1.7	4.97 ± 1.11	3.11 ± 0.59
**Tau**	4.97 ± 1.45	4.17 ± 1.35	4.96 ± 0.52	3.66 ± 0.51
**Gly**	15.81 ± 6.78	19.10 ± 3.63	16.31 ± 2.5	21.85 ± 1.32
**Lac**	3.22 ± 1.45	4.44 ± 1.01	4.7 ± 0.83	4.26 ± 1.53
**PEA**	8.08 ± 3.12	14.79 ± 8.3*	13.14 ± 2.14	15.84 ± 1.74

- The data are shown as the means ± SD.

  * – $p < 0.05$ significant differences in compared with control
